# Antimicrobial resistance patterns in UTIs in male patients with associated kidney stone disease: a descriptive study

**DOI:** 10.25122/jml-2026-0039

**Published:** 2026-04

**Authors:** Razvan-Ionut Popescu, Catalin Babita, Tudor Proca, Viorel Jinga

**Affiliations:** 1 Carol Davila University of Medicine and Pharmacy, Bucharest, Romania; 2 Department of Urology, Prof. Dr. Th. Burghele Clinical Hospital, Bucharest, Romania

**Keywords:** urinary tract infections, male, kidney stone, antibiotic resistance, PCNL, URS

## Abstract

Urinary tract infections (UTIs) and the widespread use of antibiotics are a growing concern in both the urological field and public health. UTIs affect both male and female patients similarly, with a higher incidence among individuals with excessive rates of stone formation. This study aimed to evaluate the main microorganisms and antimicrobial agents involved in urinary tract infections in men undergoing surgical procedures for urolithiasis over a 2-year period. We conducted a retrospective, single-center, observational study over 24 months, from January 2024 to December 2025. Criteria for inclusion consisted of male patients of at least 18 years old, at least one positive urine culture (>105 CFU/ml), a single bacterial agent, imaging-confirmed urolithiasis, and a history of surgical management of renal stone disease (FURS, URS, and/or PCNL). Cases that were conservatively managed or presented no evidence of urolithiasis, as well as female patients, were excluded from this study. A total of 543 patients underwent assessment. Differences were observed between the incidence of Gram-positive (155, 28.55%) and Gram-negative (388, 71.45%) microbial agents. The highest incidence was highlighted for *Escherichia coli* (32.33% of cases), followed by *Enterococcus* spp. (20.81%) and *Klebsiella* spp. (20.81%). Among the resistance rates for the main antibiotics evaluated, the highest values were observed for Levofloxacin (44.53% for Gram-negative and 55.47% for Gram-positive). This study underscores the importance of appropriate and optimized antimicrobial management, especially in patients undergoing surgery for renal stone disease, reinforcing current data.

## Introduction

Urinary tract infections (UTIs) are among the most common bacterial infections diagnosed in both outpatient and hospitalized settings. Recent data estimates that there are approximately 404.601 million new cases and more than 200,000 deaths globally, having both healthcare investment and socioeconomic impact [1]. Data published in 2021 suggest that, between 1990 and 2021, the number of UTIs increased steadily among both female and male patients of all ages [2]. Some of the latest studies demonstrate that, beyond these epidemiologic data, urinary tract infections greatly impact the population, and they are the most common outpatient infection, with a lifetime prevalence of 50-60% in young women. This incidence is rising with age, and it is presenting a doubling baseline rate in women over 65 years old [3]. Regarding hospital infections, UTIs are associated with increased mortality as a primary cause or as a complication of other pathologies [1].

Urinary tract infections exhibit well-established sex-related differences regarding epidemiology, physiology, and even clinical presentation. As a result, women are more susceptible to urinary infections due to their local anatomy and the proximity of the urethra to the rectum. Also, a shorter urethral length facilitates bacterial colonization, particularly by *Escherichia coli*, which is responsible for approximately 75–95% of uncomplicated UTIs in women [4]. There are also age-related differences in women, with sexual activity and spermicide use being important risk factors in younger women, while estrogen deficiency is more relevant in postmenopausal women [4]. In contrast to these risk factors, UTIs in men are less prevalent and, when present, are often classified as complicated infections according to most guidelines [4,5].

In contrast to the more linear age distribution found in the prevalence of UTIs in females, in males, UTIs seem to affect mostly older patients and are commonly related to obstructive pathology. The main condition associated with infections in males is benign prostatic hyperplasia (BPH), which has a continuously increasing prevalence from the 60s, affecting approximately 50–60% of men, to the 70s, where it may reach up to 80% [6]. Other important factors favoring bacterial colonization and recurrent UTIs in males are surgical instrumentation, indwelling catheters, and renal stone disease. All these causes increase antibiotic exposure, which may lead to increased resistance profiles among the causative bacteria [7].

Renal stone disease is one of the most important factors in the development of complicated urinary tract infections in both sexes, as it can cause urinary obstruction and promote bacterial adhesion and biofilm formation [8,9]. There is also a bidirectional relationship between lithiasis and infections, as bacteria such as *Proteus* spp. are often implicated in urine alkalization and crystallization, which can lead to stone formation [10].

Besides the increased prevalence of bacterial infections, the most important aspect is antimicrobial resistance (AMR), which is continuously rising globally due to the inappropriate and excessive use of antibiotics [11,12]. This is a particularly relevant phenomenon for urological clinics that often have to deal with complex cases undergoing endourological procedures, such as ureteroscopy (URS) and percutaneous nephrolithotomy (PCNL). Pre-existing infections and multidrug-resistant organisms can modify the postoperative prognosis for complications such as fever and sepsis [13,14].

Recent findings from European surveillance data have revealed an alarming increase in antimicrobial resistance among uropathogens, particularly to fluoroquinolones and third-generation cephalosporins. In Gram-negative bacteria, resistance to these antibiotic classes is frequently reported to exceed 20–40%, depending on the region and clinical setting [15,16]. Eastern Europe, particularly Romania, consistently reports some of the highest resistance rates on the continent, with *E. coli* and *Klebsiella* spp. identified as the leading pathogens associated with antimicrobial resistance.

Given that most guideline recommendations and reports on resistance rates are based on data from female patients, there is a strong need to evaluate whether resistance patterns differ in the male population. This aspect may be important for selecting the most effective empirical antibiotic strategies and avoiding unnecessary treatment.

This study aimed to evaluate the prevalence and resistance rates found in the male population undergoing endourological procedures for lithiasis at one of the most important urological centers in Romania.

## Material and Methods

This is a retrospective, observational, single-center study conducted over a 2-year period from January 1, 2024, to December 31, 2025, on male patients at one of the most important Romanian centers for the treatment of stone disease. “Prof. Dr. Th. Burghele” Clinical Hospital, Bucharest, is granted as an expert stone center by the European Association of Urology and approved for leading fellowship and master classes in conjunction with the association’s guidance.

Data from all male patients who underwent surgery for renal and ureteral stone disease in this period were analyzed based on the results of the urine test. Only positive urine cultures were included in the study.

Inclusion criteria were: male sex, age ≥18 years, positive urine culture (>10^5^ CFU/ml), single bacterial agent, imaging-confirmed urolithiasis, surgical management of stone disease (ureteroscopy, flexible ureteroscopy, and percutaneous nephrolithotomy), and availability of complete microbiological data. The exclusion criteria were: female sex, age less than 18 years, conservative (non-surgical) management, absence of urolithiasis, incomplete microbiological data, and polymicrobial or contaminated urine samples. Also, patients who underwent other procedures, such as double-J stent placement and nephrostomy tube placement, were excluded.

Surgical interventions were identified using standardized procedural coding based on the ICD-10-AM/Australian Classification of Health Interventions (ACHI) system, allowing precise categorization of endourological procedures. The following interventions were included: flexible ureteroscopy (36857-00), semi-rigid ureteroscopy (36540-00), ureteroscopic lithotripsy (36543-00), percutaneous nephrolithotomy (36654-02), as well as endoscopic lithotripsy and extraction procedures (36656-00, 36656-01, 36652-00, 36652-01).

All patients included in the study were hospitalized in a standard urology department. General information, including each patient’s demographic data (sex, age, and social status), was recorded. Following national [17] and European guidelines [18], which are updated annually, “Prof. Dr. Th. Burghele” Clinical Hospital has maintained a rational antibiotic use policy throughout the study period and to date.

In each instance, urine collection was carried out in accordance with all applicable International Safety Standards [19]. Urine samples were inoculated onto chromogenic agar and Columbia 5% blood agar using a calibrated loop delivering 0.01 mL, and the samples were incubated for 18 to 24 hours at 37°C. Samples were considered positive for UTIs if a single organism was cultivated at a concentration greater than 10^5^ CFU/mL. If the concentration was lower than 10^5^ CFU/mL, it was considered non-significant or negative and served as an exclusion criterion.

Simple descriptive statistics were generated in Microsoft Excel (version 2016, Microsoft Corporation, Redmond, WA, USA) to evaluate the data. Frequency and percentage were used to assess the relationship between the variables.

## Results

### Study population and age distribution

After applying the exclusion criteria, 543 patients diagnosed with positive urine culture and simultaneous renal or ureteral stone disease who underwent specific surgery were included in the analysis. According to age distribution, an increased prevalence could be observed in older patients (Table 1).

**Table 1:** Age distribution of the study population

	<55 years	>55 years	Total
Male	221	322	543

Distribution of male patients across age groups (<55 years and >55 years), with total counts

Regarding the overall bacterial prevalence observed in the present study, the results were consistent with the general distribution in the common population. Gram-negative bacteria were most frequently associated with stone disease (Table 2).

**Table 2:** Bacterial Gram distribution

		Male	
Group	Gram	*n*	%.
**Bacteria**	Gram-Negative	388	71.45
	Gram-Positive	155	28.55

Number and percentage of Gram-negative and Gram-positive isolates

The bacterial distribution among Gram-negative and Gram-positive cases identified *E. coli* strains as the most frequently isolated microorganism (32.23%), followed by *Enterococcus* spp. (21.92%) and *Klebsiella* spp. (20.81%). *Pseudomonas* spp. (10.5%) and *Proteus* spp. (7.92%) were confirmed to make a relevant contribution. The less frequent uropathogen was *Staphylococcus* (6.63%) (Table 3).

**Table 3:** Bacterial prevalence in the analyzed male population

		n	Male %.
Gram-Negative	*E. coli*	175	32.23
	*Klebsiella* spp.	113	20.81
	*Proteus* spp.	43	7.92
	*Pseudomonas* spp.	57	10.50
Gram-Positive	*Enterococcus* spp.	119	21.92
	*Staphylococcus* spp.	36	6.63

Number and percentage of each uropathogen species isolated

An overall analysis of antimicrobial resistance rates was conducted among Gram-negative and Gram-positive bacteria. The resistance profile highlights clear differences between the two analyzed groups. Among Gram-negative bacteria, increased resistance rates were commonly observed for antibiotics such as amoxicillin–clavulanic acid (50.52%), trimethoprim–sulfamethoxazole (46.71%), and levofloxacin (44.53%). Preserved susceptibility was observed for fosfomycin (5.42%) and amikacin (12.79%). Another important aspect is that carbapenems also showed moderate resistance rates (imipenem: 21.9%; meropenem: 29.09%).

Gram-positive bacteria showed generally high susceptibility to vancomycin (4.55%), linezolid (7.25%), and nitrofurantoin (5.97%), while significantly higher resistance rates were observed for penicillin (49.28%) and levofloxacin (50%) (Table 4).

**Table 4:** Overall antimicrobial resistance and susceptibility rates among Gram-negative and Gram-positive isolates from male patients

Antibiotic	Gram - R		Gram - S		Gram + R		Gram + S		Overall		Overall	
	*n*	%.	*n*	%.	*n*	%.	*n*	%.	*n*	%.	*n*	%.
**Amikacin**	49	12.79	334	87.21	0	0.00	8	100.0	49	12.53	342	87.47
**Amoxicillin-Clavulanic Acid**	145	50.52	142	49.48	2	22.22	7	77.78	147	49.66	149	50.34
**Ampicillin**	42	75.00	14	25.00	12	11.43	93	88.57	54	33.54	107	66.46
**Aztreonam**	41	29.71	97	70.29	0		0		41	29.71	97	70.29
**Trimethoprim-Sulfamethoxazole**	142	46.71	162	53.29	13	33.33	26	66.67	155	45.19	188	54.81
**Ceftazidime**	105	27.70	274	72.30	1	12.50	7	87.50	106	27.39	281	72.61
**Fosfomycin**	9	5.42	157	94.58	8	8.42	87	91.58	17	6.51	244	93.49
**Imipenem**	30	21.90	107	78.10	0		0		30	21.90	107	78.10
**Levofloxacin**	167	44.53	208	55.47	73	50.00	73	50.00	240	46.07	281	53.93
**Linezolid**	1	33.33	2	66.67	10	7.25	128	92.75	11	7.80	130	92.20
**Meropenem**	32	29.09	78	70.91	0	0.00	2	100.0	32	28.57	80	71.43
**Nitrofurantoin**	39	20.31	153	79.69	8	5.97	126	94.03	47	14.42	279	85.58
**Penicillin**	1	50.00	1	50.00	68	49.28	70	50.72	69	49.29	71	50.71
**Vancomycin**	0	0.0	2	100.0	5	4.55	105	95.45	5	4.46	107	95.54

Number and percentage of resistant (R) and susceptible (S) Gram-negative and positive isolates for each antibiotic tested

Regarding antimicrobial resistance rates for each bacterial species, *Escherichia coli* showed high susceptibility to last-resort antibiotics, such as carbapenems, with no resistance to imipenem or meropenem observed. Very low resistance rates were also observed for fosfomycin (1.36%), amikacin (4.68%), and nitrofurantoin (5.63%). In contrast, increased resistance patterns were observed with frequently used antibiotics, including ampicillin (69.57%), levofloxacin (44.44%), trimethoprim–sulfamethoxazole (42.86%), and amoxicillin–clavulanic acid (40%).

*Klebsiella* spp., the second most frequent Gram-negative bacteria, showed increased resistance to several classes of antibiotics, including ampicillin (84.62%), amoxicillin–clavulanic acid (65.26%), nitrofurantoin (61.36%), and meropenem (40%). Levofloxacin (45.45%), trimethoprim–sulfamethoxazole (48.04%), and ceftazidime (36.61%) also showed alarming results. Few antibiotics maintained their relative efficacy, such as amikacin (79.46%) and imipenem (72.97%) (Table 5).

**Table 5:** *Escherichia coli* and *Klebsiella* spp. antimicrobial resistance and susceptibility rates

Antibiotic	*E. coli* R		*E. coli* S		*Klebsiella* R		*Klebsiella* S	
	*n*	%.	*n*	%.	*n*	%.	*n*	%.
**Amikacin**	8	4.68	163	95.32	23	20.54	89	79.46
**Amoxicillin-Clavulanic Acid**	62	40.0	93	60.00	62	65.26	33	34.74
**Ampicillin**	16	69.57	7	30.43	22	84.62	4	15.38
**Aztreonam**	9	19.15	38	80.85	15	36.59	26	63.41
**Trimethoprim-Sulfamethoxazole**	69	42.86	92	57.14	49	48.04	53	51.96
**Ceftazidime**	31	18.24	139	81.76	41	36.61	71	63.39
**Fosfomycin**	2	1.36	145	98.64	6	40.00	9	60.00
**Imipenem**	0	0.0	47	100.0	10	27.03	27	72.97
**Levofloxacin**	76	44.44	95	55.56	50	45.45	60	54.55
**Linezolid**	1	33.33	2	66.67	0		0	
**Meropenem**	0	0.00	12	100.0	12	40.00	18	60.00
**Nitrofurantoin**	8	5.63	134	94.37	27	61.36	17	38.64
**Penicillin**	1	50.0	1	50.00	0		0	
**Vancomycin**	0	0.0	2	100.0	0		0	

Number and percentage of resistant (R) and susceptible (S) *E. coli* and *Klebsiella*

Although *Pseudomonas* spp. and *Proteus* spp. were less prevalent, their resistance trends to several important antibiotic classes provide valuable information. *Pseudomonas* spp. showed considerable resistance rates to multiple antibiotic classes, including levofloxacin (52.94%), ceftazidime (42.59%), aztreonam (40.48%), and imipenem (41.3%). Furthermore, meropenem also showed a higher rate (32.73%), suggesting a narrowing of therapeutic options even among second-line antibiotics. Amikacin remains one of the few antibiotics with a proper sensitivity rate of 71.93%.

*Proteus* spp., often considered an important contributor to stone formation, followed a similar trend, with high resistance rates to commonly used antibiotics such as trimethoprim–sulfamethoxazole (57.5%), amoxicillin–clavulanic acid (56.76%), and ampicillin (57.14%). Levofloxacin (32.56%) and nitrofurantoin (50%) demonstrate limited activity against this bacterium. Preserved sensitivity was observed for amikacin (95.35%), aztreonam, and fosfomycin (100%), as well as for carbapenems, including imipenem (85.71%) and meropenem (84.62%) (Table 6).

**Table 6:** Antimicrobial resistance and susceptibility rates of *Pseudomonas* spp. and *Proteus* spp.

Antibiotic	*Pseudomonas* R		*Pseudomonas* S		*Proteus* R		*Proteus* S	
	*n*	%.	*n*	%.	*n*	%.	*n*	%.
**Amikacin**	16	28.07	41	71.93	2	4.65	41	95.35
**Amoxicillin-Clavulanic Acid**	0		0		21	56.76	16	43.24
**Ampicillin**	0		0		4	57.14	3	42.86
**Aztreonam**	17	40.48	25	59.52	0	0.0	8	100.0
**Trimethoprim-Sulfamethoxazole**	1	100.0	0	0.00	23	57.50	17	42.50
**Ceftazidime**	23	42.59	31	57.41	10	23.26	33	76.74
**Fosfomycin**	1	100.0	0	0.0	0	0.0	3	100.0
**Imipenem**	19	41.30	27	58.70	1	14.29	6	85.71
**Levofloxacin**	27	52.94	24	47.06	14	32.56	29	67.44
**Linezolid**	0		0		0		0	
**Meropenem**	18	32.73	37	67.27	2	15.38	11	84.62
**Nitrofurantoin**	2	100.0	0	0.0	2	50.0	2	50.00
**Penicillin**	0		0		0		0	
**Vancomycin**	0		0		0		0	

Number and percentage of resistant (R) and susceptible (S) *Pseudomonas* spp. and *Proteus* spp. Isolates

Antimicrobial resistance in *Enterococcus* spp. showed a relatively preserved profile for antibiotics that have an important impact on daily clinical practice. Vancomycin (95.45%), linezolid (91.51%), nitrofurantoin (93.27%), and fosfomycin (91.4%) remained highly effective in both routine practice and hospital-administered treatment. However, significant resistance rates were observed for levofloxacin (49.11%) and penicillin (37.5%), consistent with current resistance trends for these antibiotic classes. Ampicillin remained a valuable therapeutic option, with a high susceptibility rate of 88.57%.

*Staphylococcus* spp., the least prevalent uropathogen, showed high susceptibility to several antibiotics, including linezolid (96.88%), nitrofurantoin (96.67%), and fosfomycin (100%). Similar to *Enterococcus* spp., resistance was high for penicillin (85.29%), levofloxacin (52.94%), and trimethoprim–sulfamethoxazole (41.94%) (Table 7).

**Table 7:** Antimicrobial resistance and susceptibility rates of *Enterococcus* spp. and *Staphylococcus* spp.

Antibiotic	*Enterococcus* R		*Enterococcus* S		*Staphylococcus* R		*Staphylococcus* S	
	*n*	%.	*n*	%.	*n*	%.	*n*	%.
**Amikacin**	0	0.00	8	100.0	0		0	
**Amoxicillin-Clavulanic Acid**	2	22.22	7	77.78	0		0	
**Ampicillin**	12	11.43	93	88.57	0		0	
**Aztreonam**	0		0		0		0	
**Trimethoprim-Sulfamethoxazole**	0	0.00	8	100.0	13	41.94	18	58.06
**Ceftazidime**	1	12.50	7	87.50	0		0	
**Fosfomycin**	8	8.60	85	91.40	0	0.00	2	100.0
**Imipenem**	0		0		0		0	
**Levofloxacin**	55	49.11	57	50.89	18	52.94	16	47.06
**Linezolid**	9	8.49	97	91.51	1	3.12	31	96.88
**Meropenem**	0	0.00	2	100.0	0		0	
**Nitrofurantoin**	7	6.73	97	93.27	1	3.33	29	96.67
**Penicillin**	39	37.50	65	62.50	29	85.29	5	14.71
**Vancomycin**	5	4.55	105	95.45	0		0	

Number and percentage of resistant (R) and susceptible (S) *Enterococcus* spp. and *Staphylococcus* spp.

## Discussion

### General comparison data

Recent epidemiological data suggested a higher prevalence of UTIs in older male patients, which also confirms the data obtained in the study based on patients with concomitant renal or ureteral stone disease. The age-sex relationship for these patients was described in previous studies to be associated with several factors, such as benign, prosthetic, hyperplasia, urinary retention, catheterization, institutionalization, urinary tract instrumentation, and other comorbidities that increase the incidence of developing urinary tract infection [3,20,21]. As men have totally different risk factors predisposing them to developing UTIs, there is also a difference regarding age distribution over the lifetime. Similar results were reflected by recent studies, which demonstrate that men over 60 years old, especially those hospitalized or who require urological care, present a higher risk of acquiring infections of the urinary tract [20-22].

Regarding bacterial prevalence, this study revealed a high prevalence of Gram-negative bacteria even in association with lithiasis. *Escherichia coli, Klebsiella* spp., *Pseudomonas* spp., *Enterococcus* spp., and *Staphylococcus* spp. were among the most prevalent microorganisms involved in the etiology of UTIs. *Candida* spp. may also colonize individuals receiving residential care, patients with indwelling catheters, diabetic patients, and individuals with any type of immune impairment [22]. According to regional studies, *Escherichia coli* is the leading cause of UTIs in the European population. Additionally, urease-producing bacteria, such as *Klebsiella* spp. and *Proteus* spp., play an important role in this type of infection [23]. Due to selective pressure from the overuse of various antimicrobial drugs in hospitalized patients, Gram-positive bacteria, such as *Enterococcus* spp. and *Staphylococcus* spp., are also important causes of nosocomial UTIs [24,25]. Recent data from Romania also point to *E. coli* as the primary cause of UTIs in both male [26] and female patients [27], including cases of multi-drug-resistant infections [28].

### Overall Gram-negative and Gram-positive comparison

This study highlights the predominance of Gram-negative strains among men with renal stone disease. These results confirm epidemiological data showing a similar pattern in both community-acquired and nosocomial infections. Among Enterobacteriaceae, the most common pathogen associated with these infections remains *E. coli* [3,29].

Although the present results indicate that nitrofurantoin and fosfomycin have high susceptibility rates against several bacteria, their efficacy in male patients may be limited. This antibiotic regimen may be more appropriate given its good activity against uncomplicated infections, which are more common in female patients [30,31]. In contrast to other studies, which demonstrate high sensitivity of carbapenems against Enterobacterales, the present data show moderate resistance rates for these antibiotic classes, exceeding 20%. Similar to other recent data, the present findings indicate increased resistance rates to antibiotic classes such as fluoroquinolones and β-lactams [22,31].

These alarming resistance rates in Gram-negative bacteria associated with lithiasis are supported by international results over the past 10 years in both Europe and North America, which indicate a continuous rise in resistance to fluoroquinolones, especially in *E. coli* but also in other species. Most often, this is attributed to increased antibiotic consumption and overuse [3,32].

The data obtained in the study are also aligned with the actual situation, especially after the COVID-19 pandemic, confirming that prolonged hospital stays and inappropriate antibiotic use among hospitalized patients are strongly associated with an increased risk of resistance in urinary tract infections [33,34].

These study results also align with the most recent reports on resistance rates among Gram-positive isolates. Particularly for *Enterococcus* spp., the most prevalent Gram-positive pathogen, the present results on UTIs associated with stone disease reflect a pattern similar to that observed in the general population, with maintained susceptibility to several important antibiotics, such as vancomycin, linezolid, nitrofurantoin, and fosfomycin. Increased resistance rates were also observed for fluoroquinolones and penicillin [35].

Overall, the data from this study for both Gram-negative and Gram-positive bacteria indicate alarming resistance rates to several commonly used antibiotics in patients with renal stone disease. These findings underscore the importance of evaluating local prevalence data and resistance patterns, as well as optimizing antibiotic treatment strategies.

### Gram-negative and Gram-positive resistance and sensitivity patterns

*Escherichia coli* is generally considered the most frequent pathogen identified in urine tests, and overall data reveal a high level of resistance across antibiotic classes, particularly in healthcare settings [36,37]. As previous studies strongly suggest, the present study identified alarming resistance rates to several commonly prescribed drugs, such as fluoroquinolones and β-lactams. Another important aspect highlighted by most actual scientific research concerns increased resistance to β-lactams, and global epidemiological data suggest an intensive worldwide spread in the prevalence of bacteria producing extended-spectrum β-lactamases (ESBLs) [37].

Regarding sensitivity trends for *E. coli* in patients with stone disease undergoing specific interventions, this pathogen shows a good profile for aminoglycosides, fosfomycin, and carbapenems. This indicates that these classes of antibiotics remain effective despite the rising trend in resistance rates.

*Klebsiella* spp., the second most frequently isolated pathogen, revealed important aspects regarding antimicrobial resistance by showing increased resistance rates to commonly used drugs, such as fluoroquinolones, as well as to last-resort antibiotics, such as carbapenems. These data, obtained in a specific population, appear to align with recent studies, which report an alarming trend of resistance among *Klebsiella* species [38]. The increasing resistance to last-resort antibiotics, such as carbapenems, among Enterobacterales is becoming a major concern worldwide, as carbapenemase-producing bacteria are associated with severe infections and limited therapeutic options [39].

This study also focused on other important Gram-negative pathogens, such as *Proteus* spp. and *Pseudomonas* spp., which are strongly associated with lithiasis by contributing to stone formation and affecting postoperative outcomes, particularly the risk of sepsis.

Accordingly, *Pseudomonas* spp. revealed high resistance rates to most of the analyzed antibiotic classes. The ability of this bacterium to develop resistance is well characterized in the literature and is mainly based on intrinsic mechanisms such as efflux pumps, reduced membrane permeability, and biofilm formation [40]. These specific bacterial mechanisms can severely alter the susceptibility of *Pseudomonas* spp. to aminoglycosides, fluoroquinolones, and carbapenems, making this an important threat regarding postoperative complications [40].

Additionally, *Proteus* spp., which is directly involved in stone formation and is responsible for infection-related stone disease, demonstrated increased resistance to several antibiotic classes, while maintaining a relatively good susceptibility profile to aminoglycosides and carbapenems. This pattern is also consistent with current scientific data [41].

Regarding Gram-positive bacteria, *Enterococcus* spp. was the leading pathogen in terms of prevalence and also showed interesting resistance and susceptibility patterns. Besides increased resistance to penicillins and fluoroquinolones, this pathogen showed preserved sensitivity to several important antibiotics, including nitrocefin, fosfomycin, linezolid, and vancomycin. Recent data from previous studies also identify these antibiotics as viable therapeutic options for UTIs [42,43]. As other studies suggest, *Enterococcus* spp. retains, for the moment, a preserved sensitivity profile to ampicillin, while current literature indicates an increasing trend in vancomycin resistance due to the evolution of vancomycin-resistant enterococci (VRE), which are frequently associated with prolonged hospitalization [44,45].

The least prevalent bacteria in both the Gram-negative and Gram-positive groups, *Staphylococcus* spp., exhibited adequate susceptibility to vancomycin and linezolid, consistent with recent studies demonstrating the efficacy of these drugs, especially against methicillin-resistant staphylococci [46,47].

### Relationship with stone disease and endourologic procedures

The association between urinary tract infections and urolithiasis has been previously described, and in the present study, it represents one of the main studied aspects. While certain microorganisms, particularly urease-producing bacteria, may contribute to stone formation, the primary focus remains the microbiological profile and antimicrobial resistance patterns observed in patients undergoing surgical treatment for lithiasic pathology [8,48].

Taken together, the results of the present study confirm that the microbiological spectrum and resistance patterns of uropathogens in a tertiary emergency hospital are largely consistent with those reported in the current literature. At the same time, the variability in resistance rates underscores the importance of local surveillance data in guiding empirical antibiotic therapy, particularly in settings where urgent intervention is often required.

Regarding stone formation, *Proteus* spp., a urease-producing bacterium, is classically involved due to urine alkalization and the precipitation of magnesium ammonium phosphate crystals [10]. Although it was not among the most frequent pathogens identified in the present study, its prevalence of 7.92% remains clinically important. The lithogenic potential of *Proteus* spp. is directly associated with rapid stone growth and struvite formation. On the other hand, *E. coli* and *Enterococcus* spp. have also been described as potentially lithogenic bacteria through mechanisms involving inflammation, epithelial injury, and crystal adhesion [49,50].

The relatively high prevalence of *Enterococcus* spp., *Klebsiella* spp., and *Pseudomonas* spp. is strongly associated with chronic infection and stone disease, due to their potential to form bacterial biofilms on calculi and urological devices, such as double-J stents [51]. Biofilm formation is a key factor in recurrent UTIs and significantly increases complication rates for endourologic procedures.

In clinical practice, the presence of UTI associated with stone obstructive uropathy represents one of the major concerns, according to the increased risk of developing postoperative complications such as urosepsis and septic shock [52]. The increased resistance to common antibiotics in Gram-negative bacteria revealed in the present study further complicates pre- and postoperative management. Moreover, multidrug-resistant pathogens, such as *Klebsiella* spp. and *Pseudomonas* spp., are often associated with prolonged hospitalization, increased morbidity, and a higher risk of requiring intensive care [52,53].

Another important aspect of the UTI–stone relationship is the role of biofilm-producing bacteria, even when a surgical approach is not immediately required, as they may contribute to recurrent infectious episodes until stone removal [50].

In surgical management, these microbiological characteristics affect procedures such as ureteroscopy and percutaneous nephrolithotomy. Some of the most significant postoperative risks are infectious complications, which range from fever and urinary tract infections to severe urosepsis. Three independent factors can predict post-PCNL sepsis: positive preoperative urine cultures, contaminated stones, and the presence of resistant organisms [54,55]. Furthermore, URS is associated with a higher likelihood of infectious complications, particularly in patients with pre-existing bacteriuria, prolonged surgical duration, or increased intrarenal pressure during the procedure. These factors can lead to pyelovenous and pyelolymphatic reflux of bacteria and endotoxins [56].

Furthermore, the presence of biofilm-forming organisms and antibiotic-resistant organisms, such as *Klebsiella* spp. and *Pseudomonas* spp., significantly increases the risk of postoperative sepsis and may reduce the effectiveness of perioperative antibiotic prophylaxis. Rigorous management of preoperative infections is necessary, including sterilization of urine cultures, selection of antibiotics based on local resistance profiles, and staged assessment of procedures for high-risk patients [52,53].

The present results strongly suggest that the relationship between renal and ureteral stones is both a consequence and a driver of chronic infection. This relationship highlights the need for an integrated management approach that combines surgical treatment with a comprehensive antibiotic strategy.

### Strengths and limitations

This study has many strengths. Firstly, it includes a relatively large group of male patients with confirmed urolithiasis and positive urine cultures. These cultures provide valuable data on the prevalence of urinary pathogens and antimicrobial resistance profiles. Secondly, the study analyzed cases from a high-volume tertiary referral center. The center presented a variety of cases, ranging from routine lithiasis to complex cases. The clinical accuracy and relevance of the results are enhanced by the exclusive inclusion of surgically treated patients with imaging-confirmed disease. Furthermore, the reliability and reproducibility of the results are enhanced by standardized microbiological methods and detailed profiling of resistance across different antibiotic classes.

However, several limitations must be highlighted. The single-center, retrospective design has the potential to limit the generalizability of the results and introduce selection bias. Also, this is just observational data based on the UTI-stone relation without involving other pathologies. Furthermore, prior antibiotic exposure, history of hospitalizations, or comorbidities are not accounted for in this study.

## Conclusion

The present study highlights the high prevalence of Gram-negative strains among male patients undergoing surgery for urolithiasis. Like other studies conducted in the general population, this study also reveals alarming resistance rates to commonly used antibiotics, particularly fluoroquinolones and beta-lactam combinations.

*Klebsiella* spp. and *Pseudomonas* spp. exhibited the most concerning resistance profile for several antibiotic classes in males with associated stone disease. All these findings support the notion that some-related UTIs in the male population are an important aspect, particularly because the infections encountered in this case often require optimized antimicrobial management.
